# Depression and Identity: Are Self-Constructions Negative or Conflictual?

**DOI:** 10.3389/fpsyg.2017.00877

**Published:** 2017-05-30

**Authors:** Adrián Montesano, Guillem Feixas, Franz Caspar, David Winter

**Affiliations:** ^1^Department of Personality, Assessment and Psychological Treatments, Universitat de BarcelonaBarcelona, Spain; ^2^Psychopathology and Psychotherapy Research Unit, School of Psychology, Universidade do MinhoBraga, Portugal; ^3^Department of Clinical Psychology and Psychotherapy, University of BernBern, Switzerland; ^4^Department of Psychology, University of HertfordshireHatfield, United Kingdom

**Keywords:** cognitive conflicts, implicative dilemmas, major depression, self-ideal discrepancy, self-esteem, cognitive therapy, constructivist therapy, repertory grid technique

## Abstract

Negative self-views have proved to be a consistent marker of vulnerability for depression. However, recent research has shown that a particular kind of cognitive conflict, implicative dilemma, is highly prevalent in depression. In this study, the relevance of these conflicts is assessed as compared to the cognitive model of depression of a negative view of the self. In so doing, 161 patients with major depression and 110 controls were assessed to explore negative self-construing (self-ideal discrepancy) and conflicts (implicative dilemmas), as well as severity of symptoms. Results showed specificity for the clinical group indicating a pattern of mixed positive and negative self-descriptions with a high rate of conflict. Regression analysis lent support to the conflict hypothesis in relation to clinically relevant indicators such as symptom severity, global functioning. However, self-ideal discrepancy was a stronger predictor of group membership. The findings showed the relevance of cognitive conflicts to compliment the well-consolidated theory of negative self-views. Clinical implications for designing interventions are discussed.

## Introduction

Cognitive models describe depression as involving pervasive negative views of the self, the world and the future generated and maintained by deficits and biases in information processing (e.g., Beck et al., 1979; Ingram et al., 1998). These negative beliefs are thought to be rooted in a negative self-schema which gives rise to negative thoughts that depressed patients experience in everyday life. This theory has received extensive empirical support by a large body of research concerning its core tenets, especially in relation to the pattern of low self-esteem and negative self-constructions as a marker of vulnerability for depression (e.g., Orth et al., 2009; Zeigler-Hill, 2011; Sowislo and Orth, 2013). However, as Beck recently acknowledged (Beck, 2002; Beck and Dozois, 2011), the cognitive model needs further developments and extensions. One of the main limitations of the model is that it is focused almost exclusively on patient’s negative patterns of thinking, whereas perceived positive self-attributes and, particularly, the relation between positive and negative self-constructions remains virtually unexplored.

In this article, we build on existing support for the relevance of implicative dilemmas (IDs), a type of cognitive conflict in which negative self-views (e.g., symptoms) are associated to positive aspects of the patients’ identity (see below for a detailed description). A series of studies found that IDs are significantly more prevalent in depressive participants than in controls and that they are also associated with the severity of patients’ clinical status (Feixas et al., 2014a,b; Montesano et al., 2014). Based on these results, we sought to improve our understanding of the role of these internal conflicts on the cognitive system of depressed individuals, by testing out their relevance against the classical cognitive formulation of a negative view of the self.

There are several signs indicating that the classical formulation of low self-esteem and negative self-view in depression should be extended. On the one hand, Baumeister et al. (2003) pointed out in their sound review about self-esteem that although almost every study did show that people with low self-esteem displayed higher levels of depression than people high in self-esteem, the relationship between low self-esteem and depression may conceal some influence of other variables. In addition, these authors concluded that it is far from clear that interventions aimed at boosting self-esteem are sufficient to promote positive outcomes in different contexts such as school or job performance, or interpersonal relationships.

On the other hand, although many studies have examined the content of the self-views, only a few researchers have attempted to explore their organization or structure, which is believed to be as relevant as the content itself for the maintenance of a depressive self-schema (Ingram et al., 1998). Furthermore, only few attempts have been carried out to study positive self-attributes of depressed individuals. In these studies, we can find some evidence showing that the self-system of depressed individuals is not devoid of positive content. It differs from controls in the way in which such content is organized (e.g., Space and Cromwell, 1980). Dozois and Dobson (2001a,b) found that clinically depressed patients showed significantly greater organization for negative content while the structure of positive self-defining content had fewer interconnections. However, although they studied the interconnectedness separately for positive and negative self-relevant adjectives they did not explore the interconnections between positive and negative self-perceptions neither the possible conflict among them.

A growing body of literature about intrapersonal conflict, nonetheless, has yielded promising support for its role in mental health and motivational processes (Lauterbach, 1996; Grawe, 2004; Mansell, 2005; Carey, 2008; Feixas et al., 2014b; Montesano et al., 2014). Distressing effects of inner conflicts have been observed to be influencing the onset and maintenance of a variety of psychological disorders (see Michalak et al., 2011, for a review) as well as to be associated with subjective well being and life satisfaction (Palys and Little, 1983; Little et al., 2007). Emmons and colleagues have shown that goal conflicts might generate motivational deficits, behavior inhibition, and difficulties in action control (Emmons and King, 1988; Emmons et al., 1993). As for depression, motive-goal discrepancies and inconsistencies between attitudes have been associated with higher levels of depressive symptoms beliefs (Renner and Leibetseder, 2000; Stangier et al., 2007; Püschel et al., 2011).

Despite this preliminary evidence, the role of internal conflicts in depression is yet an underresearched topic within contemporary psychotherapy and clinical psychology. More research is needed in order to clarify to what extent cognitive conflicts might play a role in the cognitive system of depressed individuals. Tracking internal conflicts is nevertheless a difficult task given their implicit nature. The relative lack of research might be due to the fact that there are not many available methods for assessing internal conflicts in clinical settings. In the last decade, Feixas and colleagues developed a procedure for a tailored assessment of internal conflicts, the so-called IDs (Feixas and Cornejo, 2002; Feixas et al., 2009).

### Implicative Dilemmas as a Way to Tap Internal Conflicts

The notion of ID draws from personal construct theory (Kelly, 1955/1991) and defines a particular type of conflict between two opposing parts of the self, two personal constructs. In personal construct theory, the cognitive system is conceived as an evolving network of bipolar personal constructs (e.g., optimistic–pessimistic) which are hierarchically interconnected. This system comprises the specific subjective way in which individuals construe their selves and the ongoing experience. However, the network of personal constructs might not necessarily reflect a logically structured cognitive system but, rather, it may comprise inconsistencies and conflicts, such as IDs.

**FIGURE 1:**
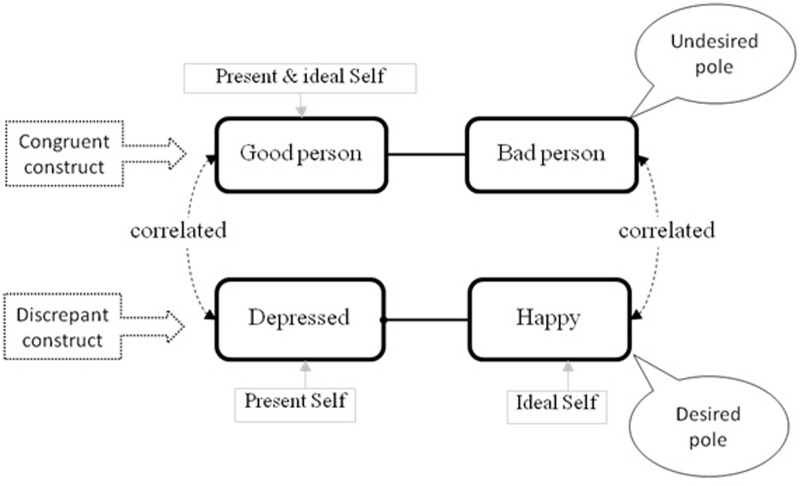
Structure of an implicative dilemma.

An ID is a conflict in which a desired change (e.g., becoming happier) simultaneously implies an unwished change on a core aspect of a client’s identity (e.g., to stop being good person and become bad person). This implication (e.g., associating being happier with becoming worse person) is based on an implicit association between the two personal constructs. The net aftermath of the clash between these two opposing forces often results in a vacillation that could paralyze clients from achieving their desired changes (e.g., symptom improvement). To further illustrate the notion of IDs, we have represented graphically its components in **Figure 1**. Basically, there are three main elements involved in and ID:

**1. Discrepant constructs** refer to the need for change. They are self-representations in which a person perceives a meaningful discrepancy between the present self and the ideal self. This type of personal construct represents perceived negative attributes, which typically indicate areas of malaise and symptoms in which a change from one pole to the opposite pole of the construct is desired. In **Figure 1**, the present self is in the pole, “depressed,” and the ideal self is in the pole, “happy.”
**2. Congruent constructs** refer to the need for continuity and personal coherence. They are constructs in which the self and the ideal self are close to each other, allocated to the same pole of the construct (e.g., good person), and therefore the person perceives congruency between the present self and the ideal self. These are mostly core constructs that are connected to personal values, beliefs, and attitudes that define the central sense of an individual’s identity (Montesano et al., 2009, 2014); therefore, a person would resist changing to avoid self-invalidation. In the example, both the actual self and the ideal self of the participant are regarded as “good person” as opposed to “bad person.”
**3. An association** between these two constructs within a patient’s personal construct system in such way that the desired change implies an unwished change, as in the example in which “becoming happier,” in turn, implies “becoming worse person.”

An ID, therefore, is detected whenever there is an association between the desired pole of a discrepant construct and the undesired pole of a congruent construct so that, in the example, being happy implies becoming a bad person. Because of that implication, this cognitive structure reflects a dilemma for the person. To put into words, “if I feel happy I risk becoming a bad person but by remaining a good person will be depressed.”

Research conducted so far on IDs has shown that they are significantly more prevalent in clinical samples than in controls (*OR* = 3.43; Montesano et al., 2015) and especially in depression. Specifically, researchers reported percentages of 68.3% of patients with a diagnosis of major depression (Feixas et al., 2014a) and 69.6% in a sample with dysthymia (Montesano et al., 2014) in contrast to roughly a third of non-clinical individuals. In addition, depressed patients tended to show a much higher number of IDs, to the extent of tripling the number in control groups. Furthermore, depressive patients with IDs presented lower levels of global functioning and more frequent history of suicide attempts and moderate to high correlations have been found between symptom level and number of IDs (e.g., Feixas et al., 2014b). All these findings lend support to the cognitive conflict hypothesis indicating that depressed people often present an underlying conflictual self-schema represented by an interconnection between positive and negative self-constructions (congruent and discrepant constructs, respectively). Accordingly, it seems that, at least for a large number of patients, it is not only negative self-perceptions, but also the conflict with positive aspects of the self, the mechanism that maintains depressive symptoms. In this study, we bring into comparison the classical cognitive theory about depression linked to low self-esteem with the cognitive conflict hypothesis, weighing the ability of the later to complement the former.

As in Beck’s cognitive model, the cognitive conflict hypothesis also considers that negative views of the self do, certainly, have a disheartening influence in depressed patients. But in our view, the main problem for changing these negative views is that often they are connected in a conflictual way with other positive views which are more central to the sense of identity in that they involve core self-constructions. In depressive patients, congruent constructs mostly concern self-perceived moral qualities (such as “sensitive,” “cares for others,” “good person”; see Montesano et al., 2014). Therefore, the opposite poles of these core constructs are really undesirable for these patients who try to avoid movement toward those poles (e.g., “insensitive,” “selfish,” “bad person”). In sum, desirable changes in patients’ negative views of the self (e.g., movement of the self from “sad” to “happy,” or from being a “worthless” to a “valuable person”), are linked in the cognitive system to undesirable changes on congruent constructs (e.g., self moving from a “good” to a “bad” person).

This approach recognizes not only the need to change (turning negative views into positive ones) but also the need to preserve core identity values and, more important, to avoid changing in an undesired direction. The conflict between these two needs might better explain the difficulties depressive patients have in developing more fully positive views of themselves, and also the difficulties therapists encounter in changing the negative views of their patients. These cognitive conflicts might also be responsible for the high rates of relapse because once negative views become more positive, these newly acquired positive views of the self can be sensed as inappropriate because of their implicit linkage with the undesired poles of the congruent constructs.

### Research Questions of the Study

As said, a recent well-controlled study (Feixas et al., 2014a) found promising evidence for the relevance of IDs indicating differences between depressed and non-depressed participants regarding prevalence of subjects with conflicts (φ = 0.33) and individual frequency of conflicts (*d* = 0.67). However, the role of negative self-views of depressed participant’s was not taken into account in the analysis. Arguably, the higher intensity and prevalence of IDs found may respond to increased negativity of depressed participants’ self-constructions. Conversely, the cognitive conflict hypothesis asserts that IDs are not an epiphenomenon of the low self-esteem but rather a particular self schema which plays a noticeable role in the cognitive system of depressive participants.

Thus, the main objective of this study was to compare the cognitive conflict hypothesis with the classical formulation of negative views of the self by performing a reanalysis of Feixas and colleagues data set. In so doing, we used data derived from the repertory grid technique (RGT; see Materials and Methods) to contrast both variables: presence of IDs as a measure of conflict versus self-ideal discrepancy as a measure of self-negativity. Concretely, in this study, we explored the following research questions: (a) Which is the predominant self-construing pattern in depressive participants as compared to controls, positive, negative, mixed, or conflictual? (b) Are cognitive conflicts a stronger predictor than negative self-views of clinically relevant measures such as level of symptoms and global functioning? (c) Are cognitive conflicts a stronger predictor of the presence of the disorder than negative self-views?

## Materials and Methods

### Participants

Data was collected from two separate groups. A depressive sample was recruited from several health care centers of the city of Barcelona (Spain) and its surrounding area. From the 233 patients who were referred to the study, a first meeting was arranged with 186 participants (the remaining 47 declined to participate or were untraceable). Nineteen of those patients were excluded because they did not meet criteria and six refused further participation in the study. The final sample consisted of 161 patients (78.3% females) meeting the following criteria: (a) 18–65 years of age, (b) diagnostic criteria for major depression (American Psychiatric Association [APA], 2000), and (c) a score of 20 or more on the Beck Depression Inventory, Second Edition (BDI-II; Beck et al., 1996). Exclusion criteria were (a) presence of bipolar, schizophrenia, or schizoaffective disorder, (b) presence of psychotic symptoms, (c) current substance abuse, and (d) organic mental disorder, brain dysfunction, or pervasive developmental delay.

The second group was composed of 110 participants (71.8% females) serving as controls and meeting the inclusion criteria of: (a) 18–65 years of age, (b) scoring less than 14 on the BDI-II (to ensure the absence of significant depressive symptoms), and (c) no presence of psychiatric or neurological illness. Participants of both groups meeting the inclusion criteria and willing to participate gave written informed consent on forms approved by local research ethics committees. After the recruitment period, the comparability of both groups was tested out. Participants’ demographic and baseline characteristics are presented in **Table 1**. As shown, no significant differences were found in regard to sex, age, marital status, and education level. On the other hand, as expected, both groups differed significantly in level of symptoms.

**Table 1:** Demographic and baseline characteristics of depressed patients and non-clinical participants.

	Depression (*n* = 161)	Control (*n* = 110)	*p*-Value
Sex (female:male)	126:35	79:31	0.29
Age (*M*; SD)	47.13; 11.31	44.51; 14.39	0.11
Married/living together (Fr; %)	93; 57.8	71; 64.5	0.32
Years of education (*M*; SD)	11.9; 2.4	12.2; 3.8	<0.001
BDI-II (*M*; SD)	36.06; 9.81	5.28; 4.36	<0.001

**M*, mean; SD, standard deviation; Fr, frequency; BDI-II, Beck Depression Inventory,Second Edition.*

### Instruments and Measures

#### Structured Clinical Interview for DSM-IV Axis I Disorders (SCID-I; First et al., 1996)

The SCID-I is a semi-structured interview which includes an introductory overview and nine modules designed to assess categorically defined DSM-IV-TR Axis I psychiatric disorders. Lobbestael et al. (2011) reported adequate test–retest reliability for major depressive disorder in clinical samples (*K* = 0.66). The output of the SCID-I was recorded for each depressed participant as the presence or absence of major depression disorder and the level of global functioning (GAF).

#### Beck Depression Inventory (Beck et al., 1996)

The BDI-II is a well-consolidated 21-item multiple-choice self-report questionnaire for assessing depressive symptom severity in clinical samples but also in general population samples. In our study, It was used to assess participants’ symptom severity and as a criterion of inclusion in both samples to control the symptom level within each group.

#### Repertory Grid Technique (Feixas and Cornejo, 2002)

The RGT is a constructivist assessment procedure which stems from Kelly’s (1955/1991) personal construct theory and was further developed by multiple researchers from a wide range of disciplines, although chiefly used in clinical studies (Saúl et al., 2012). The main strength of the RGT is that it allows an individually tailored elicitation of personal constructs. We used a paper–pencil version of the RGT which involved three different stages. First, the interviewer elicited the elements (present self, ideal self, and significant others identified by the participant) which were written to designate the columns of the grid. Secondly, personal constructs were elicited by presenting the participants with dyads of elements and asking them to identify any personally relevant way (avoiding physical attributes) in which both elements were viewed as similar and different to each other (e.g., both are “cheerful”). For each response, an opposite construct pole was also elicited (e.g., “cheerful versus gloomy”). Elicitation of constructs ended when each participant reached their saturation point. Responses anchored the two ends of a bipolar construct for which, in the third stage, participants were asked to rate all of the elements across each of the elicited constructs using a 7-point Likert scale. The resulting grid was composed of personal constructs (rows) and elements (columns) which provided a matrix of ratings for each participant. From that grid data matrix several indices of self-construing can be obtained using GRIDCOR software (Feixas and Cornejo, 2002). For the purpose of exploring the research questions of our study, the RGT stands out as very convenient instrument because it includes among its measures both, negative self-views and cognitive conflicts. Specifically, the following measures and indexes were used:

**1. Self-ideal discrepancy.** This index was used as a measure of negative self-construing, computed by the Pearson correlations between ratings of the elements “actual self” and “ideal self.” High positive correlations are usually interpreted as high self-esteem (Feixas and Cornejo, 2002). Research on the correlation between self-ideal discrepancy measured with the RGT and other global self-esteem measures such as Rosenberg’s Self-Esteem Scale has yielded coefficients ranging from 0.67 to 0.84, indicating that, although they are not the same, both measures are substantially related to each other (e.g., Arnold, 1988; Leach et al., 2001; Watson and Watts, 2001).
Although this measure may differ from other standardized traditional measures of self-negativity, it has one specific advantage: it focuses on participants’ subjective self-views. In consonance with a personal construct point of view, the valence of the self-descriptions’ content is not defined *a priori* by the researcher or by external observers, but from the participants themselves as showed by their ratings to the ideal self in each construct of their grids. Therefore, the self-ideal discrepancy index indicates areas of dissatisfaction as perceived by the participants themselves. This measure is in line with other methods that rely on a tailored semantic space model of cognition highlighting the role of self-ideal discrepancy in the regulation of depressive mood and the maintenance of negative self-schemas (Higgins, 1987; Dozois and Dobson, 2001a,b). Previous studies have already considered self-discrepancy, as measured by the RGT, as an index of negative self-construing in depression (Feixas et al., 2008), and other disorders such as fibromyalgia (Compañ et al., 2011), or eating disorders (Dada et al., 2012).
**2. Type of construct.** Based on the rating given to the self and the ideal self two kind of constructs can be identified: (a) discrepant constructs, which are those in which the ratings assigned to the element “present self” and the element “ideal self” differ by four or more points within the 7-point scale, and so they are allocated to opposite poles. They represent areas of self-dissatisfaction associated to negative views of the self, and (b) congruent constructs, in which there exists a coincidence or a maximum of one point of discrepancy between the “present self” and the “ideal self” elements and, therefore, are allocated in the same pole of the construct. Congruent constructs represent areas of self-satisfaction often connected to personal values and beliefs.
Although congruent and discrepant constructs are part of IDs they have never been used as measures for the specific assessment of self-positivity and self-negativity, respectively. We use them to explore between-group differences in patterns of self-construing. This measure was not included in the previous study by Feixas et al. (2014a).
**3. Cognitive conflicts.** This is a categorical variable indicting presence or absence of conflict. As explained in the Section “Introduction,” an IDs is composed by a combination of a congruent construct and a discrepant construct. Specifically, IDs are detected if the Pearson’s correlation between the ratings to all elements of congruent and discrepant constructs are equal to or higher than 0.35, so that the desirable pole of the discrepant construct is associated with the undesirable pole of the congruent construct (see **Figure 1**; Feixas et al., 2009). The rationale for using a criterion of 0.35 is based on Cohen’s guidelines (Cohen, 1988) about the interpretation of Pearson’s correlation strength.

Studies testing the reliability of patterns of construct relationships tend to yield coefficients which fall within the range of 0.60 to 0.80 (see Fransella et al., 2004 for a review). Feixas et al. (1992) looked at the stability of the self-ideal discrepancy over three time intervals obtaining reliability coefficients ranging from 0.78 to 0.94 (*p* < 0.001). Caputi and Keynes (2001) also found considerable stability of the relationships between self and ideal self, with coefficients ranging from 0.61 to 0.81.

### Procedure

During a period of 3 years, GPs and mental health professionals working in various public health services referred depressed candidates to the study. For each patient, two independent and specifically trained master-level students administered the above-mentioned instruments in face-to-face interviews at the health centers from which each patient was referred. The assessment process consisted of two interviews of approximately 2 h. Especial attention was paid to the outcome of the SCID-I and the BDI-II to determine if patients met inclusion criteria during the first interview. If so, a second appointment was scheduled to complete the RGT. Each pair of evaluators was supervised by a research assistant before and after every assessment to ensure adequate adherence to the study protocol.

Recruitment of control group participants was made through two different channels. On the one hand, some agreements were made between the research group and an array of civic associations in order to obtain volunteers. In exchange, free psycho-educational talks were offered for their participation in the study. On the other hand, a call was made among graduate and undergraduate students to refer non-clinical participants (friends and relatives) for the study. Non-depressed participants were also evaluated with the BDI-II to rule out likely non-diagnosed depressive participants. Then, if necessary, a second appointment was scheduled to complete the RGT assessment process. Participants from both groups provided informed consent at the first meeting.

Data collected from RGT was analyzed with the software GRIDCOR v. 4.0 (Feixas and Cornejo, 2002) to obtain the self-construing indices. The Statistical Package for Social Sciences version 21 (IBM Corporation, Armonk, New York, United States) was used for further regression analyses.

## Results

### Which is the Predominant Self-Construing Pattern in Depressive Participants?

In order to determine which kind of self-construing pattern prevailed in both samples, a Chi-squared test was performed to compare four different conditions: (a) participants presenting only congruent constructs, (b) only discrepant constructs, (c) both congruent and discrepant constructs without IDs, and (d) at least one ID (see **Table 2**). While the clinical sample presented significantly more participants with IDs, there were more participants presenting only congruent constructs in the control group [χ^2^ (3, *N* = 271) = 65.91; *p* < 0.001] with a large effect size (Cramer’s *V* = -0.33). A noteworthy outcome is that only two participants (1.2%) from the clinical sample (none of the controls) showed a grid configuration with only discrepant constructs (see **Table 2**), meaning that a completely negative self-construing pattern was very rare. A significant difference was also found [*t*(305.5) = 5.80, *p* < 0.001; *d* = 0.66] indicating more congruent (*M* = 8.20, SD = 4.80) than discrepant constructs (*M* = 5.38, SD = 3.85) within the clinical sample, that is, more positive than negative self-constructions.

**Table 2:** Comparison between samples in type of self-construing pattern.

		Only congruent	Only discrepant	Both but no dilemmas	Implicative dilemmas
Depression	Fr (%)	8 (5)	2 (1)	41 (25.5)	110 (68.3)
	Sr	-8.0^∗^	1.2	1.0	5.5^∗^
Control	Fr (%)	50 (45.5)	–	22 (20)	38 (34.5)
	Sr	8.0^∗^	-1.2	-1.0	-5.5^∗^

*Fr, frequency; Sr, adjusted standardized residuals. ^∗^Sr > ± 1.96.*

### Are Cognitive Conflicts a Stronger Predictor than Negative Self-Construing of Clinically Relevant Measures?

A series of regression analyses were performed to assess the capacity of IDs versus self-ideal discrepancy to predict levels of symptoms (BDI-II), and global functioning (GAF). The correlations between actual and ideal self were transformed into Fisher’s *z* scores for the regression analyses. Preliminary analyses were conducted to ensure no violation of the assumptions of normality, linearity, multicollinearity, and homoscedasticity. First, standard multiple regression was used to estimate the potential of the variables (self-ideal discrepancy and presence of conflict) to predict levels of BDI-II. The total variance explained by the model as a whole was 35% (*R*^2^), *F*(2,268) = 73.09, *p* < 0.001. Both variables were statistically significant, with self-ideal discrepancy recording a higher β value (β = -0.53, *p* < 0.001) than presence of conflict (β = 0.13, *p* = 0.013). A second standard multiple regression was performed to predict levels of GAF within the clinical sample. The model as a whole explained 9% of the variance, *F*(2,158) = 7.58, *p* < 0.001, with both predictors making a statistically significant and similar contribution (conflict β = 0.19, *p* < 0.05; self-ideal discrepancy β = 0.21, *p* < 0.05).

### Are Cognitive Conflicts a Stronger Predictor of the Presence of the Disorder than Negative Self-Views?

A logistic regression was performed to assess the predictive capacity of self-ideal discrepancy and presence of IDs on the likelihood of belonging to the clinical sample. The full model was statistically significant with no interaction effects, χ^2^ (2, *N* = 271) = 103.61; *p* < 0.001, and explained between 31.8% (Cox and Snell *R*^2^) and 43% (Nagelkerke *R^2^*) of the variance, correctly classifying 76% of cases. As shown in **Table 3**, both self-ideal discrepancy and presence of IDs made a unique statistically significant contribution to the model. The strongest predictor was self-ideal discrepancy, recording an odds ratio of 0.4.

**Table 3:** Results of the logistic regression predicting belonging to the depression or control groups.

	*B*	SE	Wald	*df*	Significance	Odds ratio	95% CI for odds ratio	
							Lower	Upper
Presence of IDs	0.78	0.31	6.28	1	0.021	2.18	1.18	4.02
Self-ideal discrepancy	-3.32	0.47	50.07	1	0.000	0.04	0.01	0.09
Constant	0.89	0.29	9.55	1	0.002	2.43		

**FIGURE 2:**
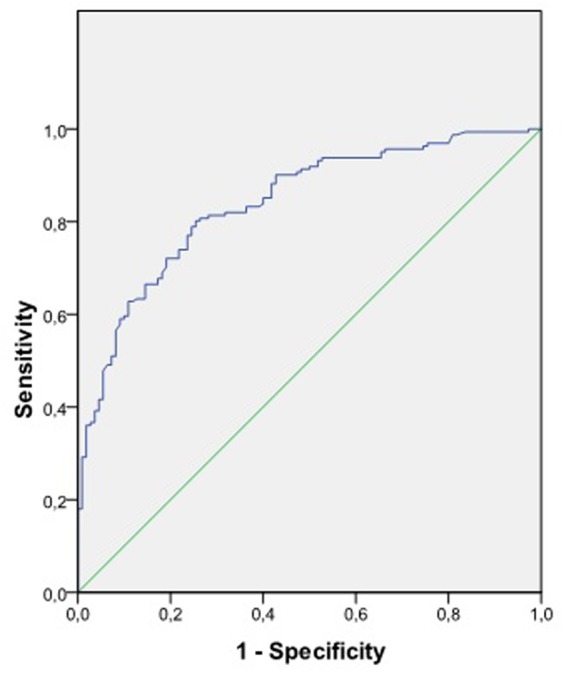
Receiver operating curve to test regression model accuracy.

A receiver operating characteristic curve was constructed to test the accuracy of the logistic regression model for predicting group membership (see **Figure 2**). The area under the curve equaled 0.85 (SE = 0.02, *p* < 0.001; 95% CI = 0.80–0.89), indicating that the regression model was accurate, and prediction was significantly different from random assignment. Fixing the cutoff point at 0.54 and, thus, maximizing the false positive to false negative ratio, the model showed 80.7% sensitivity and 73.8% specificity.

## Discussion

This study appraised the relative importance of positive and negative self-representations and their conflictive organization in participants with a major depressive disorder. The overarching hypothesis was that cognitive conflicts would better explain the personal constructions of depressive patients than negative self-views. In order to do so, we first explored the patterns of self-construing in a sample of major depressive patients as compared to a non-depressed group and then evaluated their capacity to predict several clinical indicators.

With regard to the valence of self-constructions, we found that 93.8% of the clinical participants showed a mixed pattern of positive and negative self-perceptions, 5% presented with exclusively congruent constructs in their grids, and only two participants (1.2%) had a “pure” self-negative pattern. These results are in line with the literature showing that the self-system of depressed individuals is not devoid of positive content. Indeed, clinical participants of our study displayed significantly more positive constructions of the self (congruent constructs). It seems that the most prevalent pattern is a mixture of positive and negative self-views. However, results also indicated that up to the 73% of depressed participants with mixed patterns presented with conflictual configurations in their cognitive-system. In contrast to controls, there was a large effect size in the presence of conflicts, being this the most salient between-groups difference.

With regard to the ability of conflicts to predict several clinical indicators, results were mixed. When the variables (self-ideal discrepancy and presence of conflict) were entered in the model for predicting level of depressive symptoms, self-ideal discrepancy was the best predictor. The inverse relationship between self-esteem and depression has been extensively validated (see Silvia and Eddington, 2012; Sowislo and Orth, 2013) whereas the nature of the relationship between the presence of conflict and symptoms still remains unclear. Although some studies found high correlations (Montesano et al., 2014), most have reported a medium correlation coefficient, suggesting that the relationship between these variables may not be linear. Despite this result did not confirm our prediction, it was not totally a surprising finding that self-ideal discrepancy was most predictive of symptom severity. However, the presence of IDs also uniquely contributed to the model. Thus, we may contemplate number of IDs as a variable playing some secondary role with respect to symptom severity.

The relevance of the conflict hypothesis was also tested out by predicting the level of global functioning. In this case, both, self-ideal discrepancy and conflict, made a similar contribution to the model. The GAF entails a broader assessment of the severity of the disorder than the BDI-II by taking into account several indicators such as social, occupational and psychological impairment (American Psychiatric Association [APA], 2000). Although our results suggested that cognitive conflicts might be associated to the functional limitations of depression, the low percentage of total variance explained by the model (9%) indicated that other mechanisms must be involved. Suffice it to say that, for instance, employment status or physical pain may have greater influence on GAF scores. The degree to which complexity of clinical situations is somehow linked to a higher frequency of dilemmas deserves the attention of further research.

An additional outcome supporting the cognitive conflict hypothesis arose when generating a model for classifying participants into clinical or control groups. *R*^2^ values indicated that the model as a whole was able to explain a respectable level of variance, and a receiving operating curve confirmed its accuracy, yielding adequate levels of sensitivity and specificity for predicting group membership. In the resultant equation, the presence of IDs emerged with less predictive power than self-ideal discrepancy, but still made a unique significant contribution to the model. It is important to note that IDs cannot be seen as completely independent from self-ideal discrepancy insofar as IDs require the presence of discrepant constructs for which there is a substantial difference between self and ideal self. However, the model indicated that even though it is true that negative self-views were highly prevalent, IDs were not an epiphenomenon but an independently relevant self-schema to understand the nature of depressive patients’ cognitive system.

In sum, depressed participants in this study showed a mixed pattern of positive and negative self-perceptions with a high rate of conflict. When comparing the ability of the variables to explain clinically relevant measures, self-ideal discrepancy was a better predictor of the severity of symptoms, and presence of the disorder but not of global functioning. Nevertheless, both indices significantly contributed on each model. A global evaluation of the results, therefore, led us to conclude that cognitive conflicts were not better predictors for depression than negative self-construing, but still they showed a certain level of predictive power. Certainly, in the light of our results, IDs might complement the well-established evidence for a negative self-schema in depression by recognizing not only the necessity to change negative self-constructions but the need to conserve the coherence with positive self-defining attributes.

This need to preserve self-coherence has also been highlighted by other theories like self-verification (Swann, 2012) or self-regulation (Vohs and Baumeister, 2011), as well as by certain therapeutic models such as coherence therapy (Ecker et al., 2012) or some systemic approaches (e.g., White, 2007). However, no operational procedures have been created so far to detect implicit conflict configurations between negative and positive self-constructions. Thus, the concept of ID provides an innovative contribution not only for the understanding of cognitive structures underlying pathological processes but also for therapy interventions inasmuch as they might be amenable of therapeutic work (see Feixas et al., 2013).

A core strategy in cognitive therapy for depression consists of identifying depressed patients’ negative self-representations, and directly challenging patients’ beliefs about themselves (Beck et al., 1979). However, the cognitive conflict hypothesis suggests that such an intervention would increase its efficiency by taking into account the possible positive attributes related to symptoms and negative self-perceptions. In doing so, the intervention could become more acceptable to patients and, thus, better help in therapeutic adherence and in decreasing the chance of relapse and symptom persistence. A central aspect of this strategy would be to integrate the desired change with the global self-system and, especially, the preservation of the sense of identity. Even though cognitive therapy has been shown to be effective for recovery in the treatment of depression (e.g., Beck and Dozois, 2011), relapse and recurrence still continue to be a prickly challenge to deal with. Further longitudinal research might explore the relevance of cognitive conflicts to predict relapse and recurrence.

There are, nonetheless, some limitations that need to be acknowledged and taken into account when looking at clinical and theoretical implications of the results. First, the cross-sectional design of the study precluded casual analysis of the relationship between variables. Secondly, as inclusion criteria we stablished BDI-II cut-offs for the clinical (above 19) and the control (below 14) participants to avoid undiagnosed depression in control participants and to ensure between-group differences in depression scores. However, these criteria also affect the generalizability of the results given that they exclude people showing scores between 14 and 19 corresponding to mild levels of depressive symptoms.

Finally, despite the evidence of the correlation between standard measures of self-esteem and the self-ideal discrepancy, it would be necessary to include in further research an explicit well-established measure of self-esteem (e.g., Rosenberg) to increase the generalizability of the findings. Ideally, given that self-esteem is a complex construct that entails several dimensions, the measurement of both implicit and explicit level of self-esteem would allow measuring not only the level but also the soundness and stability of participants’ self-esteem and so the models tested out in the regression analyses would provide more accurate outcomes. In this sense, another limitation of the study is the absence of traditional standardized questionnaires measuring self-negativity and other specific markers of cognitive vulnerability to depression. Future studies should consider combining the assessment of different levels of cognitive markers such us self-schemata, dysfunctional attitudes, or automatic thoughts to better depict the role of conflicts on the cognitive system of depressed individuals. However, the assessment of self-esteem by means of the RGT was both a limitation and an advantage. Compared to traditional measures of self-esteem, the RGT assesses the self-ideal discrepancy on the basis of meaningful terms, their personal constructs, elicited by the participants themselves.

## Author Contributions

AM and GF substantially contributed to the conception and design of the work as well as to the analyses and interpretation of the results. AM wrote the draft of the study and GF, FC, and DW reviewed the text providing relevant intellectual and technical support.

## Conflict of Interest Statement

The authors declare that the research was conducted in the absence of any commercial or financial relationships that could be construed as a potential conflict of interest.
